# Effects of active exergames on physical performance in older people: an overview of systematic reviews and meta-analysis

**DOI:** 10.3389/fpubh.2024.1250299

**Published:** 2024-04-09

**Authors:** Jordan Hernandez-Martinez, Francisco Ramos-Espinoza, Cristopher Muñoz-Vásquez, Eduardo Guzman-Muñoz, Tomas Herrera-Valenzuela, Braulio Henrique Magnani Branco, Maria Castillo-Cerda, Pablo Valdés-Badilla

**Affiliations:** ^1^Department of Physical Activity Sciences, Universidad de Los Lagos, Osorno, Chile; ^2^Programa de Investigación en Deporte, Sociedad y Buen Vivir, Universidad de los Lagos, Osorno, Chile; ^3^Doctoral Program in Psychology, Faculty of Health Sciences, Universidad Católica del Maule, Talca, Chile; ^4^Department of Health, Programa de Prevención y Rehabilitación Cardiovascular, CESFAM Dr. Juan Carlos Baeza, San Clemente, Chile; ^5^Faculty of Health, School of Kinesiology, Universidad Santo Tomás, Talca, Chile; ^6^Faculty of Health Sciences, School of Kinesiology, Universidad Autónoma de Chile, Talca, Chile; ^7^Department of Physical Activity, Sports and Health Sciences, Faculty of Medical Sciences, Universidad de Santiago de Chile (USACH), Santiago, Chile; ^8^Graduate Program in Health Promotion, Cesumar University (UniCesumar), Maringá, Brazil; ^9^Department of Physical Activity Sciences, Faculty of Education Sciences, Universidad Católica del Maule, Talca, Chile; ^10^Sports Coach Career, School of Education, Universidad Viña del Mar, Viña del Mar, Chile

**Keywords:** exergaming, virtual reality, physical functional performance, postural balance, muscle strength dynamometer, aged

## Abstract

This overview assessed the available body of published peer-reviewed systematic reviews and meta-analyses related to the effects of active exergames compared with active/passive control on physical performance outcomes in older people. The methodological quality and certainty of evidence were assessed using PRISMA, AMSTAR 2, and GRADE. The protocol was registered in PROSPERO (code: CRD42023391694). The main outcomes indicate that 4,477 records, five systematic reviews, and 10 meta-analyses were included. The AMSTAR-2 reported six meta-analyses with high methodological quality, four moderate quality, two systematic reviews with low quality, and three very-low quality. Meta-analysis was performed on balance using the Berg Balance Scale (BBS) and Timed Up-and-Go (TUG) tests, on cardiorespiratory fitness using the 6-min walk test, and on upper and lower limbs muscle strength using the handgrip strength, knee extension, and 30-s chair stand tests. Significant differences in favor of the active exergames groups concerning active/passive groups were reported in BBS (SMD = 0.85; 95% CI = 0.12–1.58; I^2^ = 96%; *p* = 0.02), TUG (SMD = 1.44; 95% CI = 0.71–2.16; I^2^ = 97%; *p* < 0.0001), and 30-s chair stand test (SMD = 0.79; 95% CI = 0.33–1.25; I^2^ = 88%; *p* = 0.0008). However, no significant differences were reported in favor of the active exergames groups in 6-min walk (SMD = 0.93; 95% CI = −0.64 to 2.50; I^2^ = 95%; *p* = 0.24), handgrip strength (SMD = 0.67; 95% CI = −0.04 to 1.38; I^2^ = 84%; *p* = 0.06), and knee extension tests (SMD = 0.20; 95% CI = −0.05 to 0.44; I^2^ = 95%; *p* = 0.12) compared to active/passive control. However, it was impossible to perform a meta-analysis for the variables of walking speed as a fall risk due to the diversity of instruments and the small number of systematic reviews with meta-analysis. In conclusion, interventions utilizing active exergames have shown significant improvements in the static and dynamic balance and lower limb muscle strength of apparently healthy older people, compared to control groups of active/inactive participants, as measured by BBS, TUG, and 30-s chair stand tests. However, no significant differences were found in the 6-min walk, HGS, and knee extension tests.

**Systematic review registration:**

PROSPERO, CRD42023391694.

## 1 Introduction

The aging process leads to different physical changes in older people, such as a 59% decrease in balance (1), 73% loss of walking speed (2), 35% lower mobility (2), 30% decrease in muscle strength (3), and 80% increased fall risk (4). Elements that together increase frailty and reduce functional independence (5) affect the quality of life in older people (6).

On the contrary, regular physical activity practice has reported improvements of 54% in physical fitness in older people after interventions that include balance, resistance training, and endurance exercises (7), a fact that aligns with international physical activity recommendations that indicate performing between 150 and 300 min of moderate-intensity physical activity or 75–150 min of vigorous-intensity physical activity a week (8), which include at least two weekly sessions of resistance training in older people (9). However, the COVID-19 pandemic has forced a rethinking of physical activity strategies for the general population (10), particularly in older people (11). Thus, performing physical activity in small spaces while maintaining distance and safety in practice has become indispensable (10, 11); in such a context, active exergames are presented as alternatives that meet these requirements and are also entertaining for older people (12, 13).

The active exergames can be developed in health, community, and sports centers or a homeroom individually or in groups using a screen (14) through active exergames that involve the movement of the whole body, similar to the real world with or without the use of a controller allowing the movements to be executed within a reduced space and interacting with the game scenario (14). Conversely, the cost to acquire a game console is affordable and ranges from US$100 to US$299, while annual healthcare costs amount to US$2,000 in the United States of America (15) and €2,337 in England, according to the National Health Service of the Kingdom United (16). The most popular consoles and active exergames used by older people are Nintendo Wii® with the Wii sports, Wii balance, and Wii Fit games (17–19); Microsoft’s Xbox Kinect^®^ with the Kinect Sports, Adventure, and Your Shape games (18, 19); and Sony’s PlayStation Move^®^ with the Sports Champions Move game (13).

Specifically, Wii Fit has demonstrated a 34% increase in lower limb muscle strength, a 23.6% increase in balance, a 35.1% reduction in fall risk, and an 8% lower risk of frailty in Hong Kong in older people compared to patients undergoing a 6-week conventional balance training intervention (20). Similarly, using a 5-week Xbox Your Shape intervention, Yang et al. (21) reported a 14.3% improvement in dynamic balance and a 16% improvement in static balance in Taiwan’s older people over a conventional balance intervention. Another study by Hernandez-Martínez et al. (22) reported notable enhancements in physical performance among older Chilean people following an 8-week Xbox Kinect Sports intervention. The researchers observed a 4.1% increase in walking speed, an 8.5% improvement in the timed up-and-go test (TUG), and a remarkable 16% enhancement in lower limb muscle strength. These findings are in line with a study conducted by Yu et al. (23) on Taiwanese older people, who experienced a 15.6% increase in lower limb muscle strength and a 5.4% improvement in cardiorespiratory fitness after engaging in a 10-week intervention utilizing Xbox Kinect Adventure. These results collectively indicate the potential benefits of utilizing Xbox Kinect-based interventions to promote physical fitness and performance among older populations.

The results that active exergames report on the physical functional and physical fitness in older people (23, 24), added to the increase of studies with high-quality design (randomized controlled trials), have allowed the development of several systematic reviews with and without meta-analysis that synthesize this evidence (19, 25–28). However, to the best of our knowledge, a study that condenses all these systematic reviews into a single document has not yet been published. In this sense, the main aim of this overview was to assess the available body of published peer-reviewed systematic reviews and meta-analyses related to the effects of active exergames compared with active/passive controls on physical performance outcomes in older people.

## 2 Materials and methods

### 2.1 Protocol and record-keeping

This overview followed the Preferred Reporting Items for Systematic Review and Meta-Analysis Protocols (PRISMA) guidelines (29). The protocol was registered in PROSPERO (International Prospective Register of Systematic Reviews; code: CRD42023391694).

### 2.2 Eligibility criteria

The inclusion criteria for this overview were original peer-reviewed systematic reviews and meta-analyses without any language or publication date restrictions, published up to 23 February 2024. Excluded records were conference abstracts, books, book chapters, editorials, letters to the editor, trial records, case studies, and trials. In addition, the population, intervention, comparator, outcome, and study design (PICOS) framework were followed to incorporate studies into an overview (see Table 1).

**Table 1:** Selection criteria used in the overview.

Category	Inclusion criteria	Exclusion criteria
Population	Older people with a mean age of 60 years or more, according to the World Health Organization WHO (2021), are healthy and without distinction of sex.	People with a mean age of fewer than 60 years old.
Intervention	Systematic reviews and meta-analysis of interventions with active exergames (i.e., Wii sports, balance and fit, Kinect Sports, Adventure and Your Shape, Sports Champions Move) of 2 weeks or more.	Systematic reviews and meta-analyses of interventions that do not use active exergames as an intervention.
Comparator	Systematic reviews with and without meta-analysis with control groups with (other than active exergames) or without supervised physical activity.	Absence of control groups.
Outcomes	At least one physical performance assessment (i.e., handgrip strength, walking speed, fall risk, Berg Balance Scale), pre- and post-intervention.	Lack of reference and/or follow-up data.
Study design	Systematic reviews or meta-analyses with the inclusion of studies using an experimental design (randomized controlled trial and non-randomized controlled trial) with pre- and post-assessments.	Systematic reviews or meta-analyses with cross-sectional, retrospective, and prospective studies.

### 2.3 Information search process and databases

The search process used seven databases: PubMed, Web of Science (core collection), Scopus, CINAHL, Cochrane Library, MEDLINE, and Psychology and Behavioral Sciences (EBSCO). Medical Subject Headings (MeSH) from the National Library of Medicine of the United States of America were used as free language terms related to active exergames, physical performance, and older people. The search string used was as follows: (“exergames” OR “exergaming” OR “active video games” OR “virtual reality” OR “wii” OR “Kinect” OR “play station”) AND (“physical function” OR “physical performance” OR “physical fitness” OR “functionality” OR “functional Independence” OR “functional dependency” OR “functional mobility” OR “health condition” OR “falls” OR “fall risk” OR “risk of fall” OR “falling risk” “balance” OR “static balance” OR “dynamic balance” OR “walking speed” OR “gait speed” OR “mobility” OR “strength” OR “muscle strength” OR “upper body strength “OR “lower body strength” OR “muscle power”) AND (“elderly” OR “older adults” OR “older people” OR “older subject” OR “aging” OR “ageing” OR “aged”).

### 2.4 Study selection and data collection process

Studies were exported to the EndNote reference manager (version X9. Bld.12062, Clarivate Analytics, Philadelphia, PA, United States). Two authors (JHM and PVB) independently searched, removed duplicates, reviewed titles and abstracts, and analyzed full texts. The process was repeated for searches within reference lists and suggestions provided by external experts. Subsequently, potentially eligible studies were reviewed in full text, and the reasons for excluding those not meeting the selection criteria were reported.

### 2.5 Assessment of methodological quality

The methodological quality of the selected studies was assessed using the AMSTAR-2 (A Measurement Tool to Assess Systematic Reviews 2) (30). This instrument is composed of 7 domains, which are as follows: (*i*) protocol recorded prior to the review; (*ii*) adequate literature search; (*iii*) justification of excluded studies; (*iv*) risk of bias of individual included studies; (*v*) appropriate meta-analytic methods; (*vi*) consideration of risk of bias in the interpretation of review results; and (*vii*) assessment of the presence and likely impact of publication bias. Sixteen items are included to rate the methodological quality of the reviews according to the following confidence criteria (30): (*i*) high: no critical weaknesses and up to one non-critical (the systematic review provides an accurate and complete summary of the results of the available studies); (*ii*) medium: no critical weaknesses and more than one non-critical weakness (although, if there are many, a low confidence could be justified): the systematic review has weaknesses, but no critical flaws, being able to provide an accurate summary of the results of the available studies; (*iii*) low: up to one critical weakness, with or without non-critical weaknesses: the systematic review may not provide an accurate and complete summary of the available studies; and (*iv*) critically low: more than one critical weakness, with or without non-critical weaknesses: the systematic review is not reliable. Two authors (JHM and CMV) independently assessed the quality of the reports using PRISMA in a 27-item checklist (29), and a third author (PVB) acted as referee for borderline cases, which were then validated by another author (THV). Each item was assessed according to whether it was reported and received 1 point for full reporting, 0.5 points for partial reporting, and 0 points for not reporting. Less than 15 points indicate relatively severe reporting defects, between 15 and 21 points indicate some reporting defects, and between 21.5 and 27 points indicate a relatively complete report.

### 2.6 Certainty of evidence

Using the GRADE scale, the degree of certainty of evidence was evaluated, and it was determined whether the studies’ degree was high, moderate, low, or very low (31). Due to the inclusion of studies with experimental designs (randomized controlled trials and non-randomized controlled trials), all analyses began with a grade of high certainty and were downgraded if there were issues with consistency, precision, directness of the results, or risk of publication bias (31). Two authors (JHM and CMV) independently assessed the studies, and any discrepancies were resolved through consensus with a third author (PVB). The criteria for downgrading the certainty of evidence were as follows: (*i*) limitation of included studies: one level of a downgrade if 25% or more of the included articles had a high risk of bias as assessed by AMSTAR-2; (*ii*) inconsistency: one level of downgrade was applied if there was high heterogeneity (I^2^ ≥ 90%); (*iii*) indirectness: One level of downgrade was applied if there were differences between participants, interventions, outcome measures, or indirect comparisons; (*iv*) imprecision: one level of downgrade was considered if there was a wide confidence interval, crosses the line of no effect, and/or small sample size (*n* < 300); (*v*) risk of publication bias: one level of downgrade was applied if there was asymmetry in the doi plot.

### 2.7 Data synthesis

The following data were obtained and analyzed from the selected studies: (*i*) author and year; (*ii*) study design (systematic reviews and/or meta-analysis); (*iii*) baseline health status of the sample; (*iv*) the number of studies and participants in the intervention and control groups; (*v*) mean age of the sample; (*vi*) activities performed in the consoles, active exergames, and control groups; (*vii*) training volume (total duration, weekly frequency, and time per game); (*viii*) physical performance data collection instruments; (*ix*) main outcomes of the systematic reviews and/or meta-analysis; (*x*) quality assessment; and (*xi*) PRISMA score.

### 2.8 Summary measures for meta-analysis

A meta-analysis was performed to explore the effect of different systematic reviews or meta-analyses on the same outcome; this was done using data from systematic reviews or meta-analysis reports included in the overall review. The pooled effect that was calculated is the standardized mean difference (SMD), 95% confidence interval (95% CI), and the inverse variance weighting method with a random effect was used. When only the median and extreme or quartiles were reported in systematic reviews or meta-analyses whose mean and standard deviation (SD) could not be obtained from systematic reviews, neither the mean nor the SD was estimated because the sample size is too small for an accurate estimated value (32). Heterogeneity was quantified by I^2^, where a value >50% indicates substantial heterogeneity (33). Low-quality systematic reviews were excluded from the sensitivity analysis, following the recommendations of previous overviews (33). These were meta-analyzed using RevMan 5.4 following the findings of previous studies (34, 35). Finally, publication bias was measured using Egger’s regression asymmetry test to assess small study effects as proposed by Sterne et al. (36). Statistical analyses were performed with StataMP, version 17 (StataCorp, College Station, TX, United States).

## 3 Results

### 3.1 Study selection

**Figure 1:**
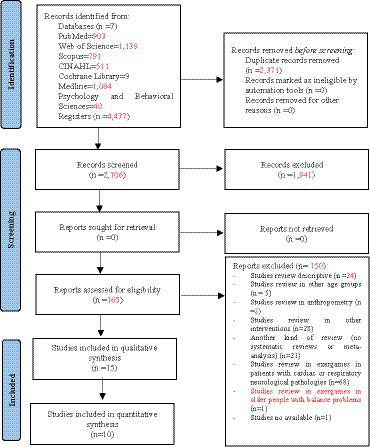
Flowchart of the review process. Based on the PRISMA-P recommendations (29).

Figure 1 details the process of searching for systematic reviews and meta-analyses. In the identification phase, a total of 4,477 records were found. Subsequently, duplicates were eliminated, and studies were filtered by selecting title, abstract, and keywords, resulting in 2,106 references. A total of 165 systematic reviews and meta-analysis were included in the next phase of analysis: 24 descriptive systematic reviews, five systematic reviews conducted in people with a mean age less than 60 years, two systematic reviews that reported anthropometric results, 28 systematic reviews of studies with interventions without active exergames, 21 studies that were not systematic reviews (other types of review), 68 systematic reviews of active exergames in patients with cardiac or respiratory neurological pathologies, one review of the study in exergames in older people with balance problems, and one systematic review because the text was inaccessible (the authors of the systematic review were contacted requesting a copy of their manuscript, estimating 30 days as maximum response time). After this process, the total number of systematic reviews and meta-analysis that met all the selection criteria was 15 (17–19, 25–28, 37–44).

### 3.2 Methodological quality

According to the AMSTAR-2, the mean score of the selected systematic reviews and meta-analysis was 12.6 points; in particular, six meta-analyses obtained a high quality with values between 15 and 16 points (18, 28, 37, 38, 41, 43), four meta-analysis obtained a moderate quality with values between 13 and 14 points (17, 26, 27, 42), two systematic reviews were of low quality with values between 10 and 11 points (19, 44), and three systematic reviews achieved a critically low quality located between 7 and 8 points (25, 39, 40). These results can be seen in Table 2.

**Table 2:** Methodological quality.

Questions	References														
	Afridi et al. (25)	Chen et al. (26)	Corregidor-Sánchez et al. (18)	Corregidor-Sánchez et al. (17)	Fang et al. (37)	Ge et al. (19)	Janhunen et al. (38)	Laufer et al. (39)	Liu et al. (27)	Molina et al. (40)	Pacheco et al. (28)	Suleiman-Martos et al. (41)	Taylor et al. (42)	Viana et al. (43)	Zheng et al. (44)
1. Did the research questions and inclusion criteria for the review include the components of PICO?	Yes	Yes	Yes	Yes	Yes	Yes	Yes	Yes	Yes	Yes	Yes	Yes	Yes	Yes	Yes
2. Did the review report contain an explicit statement that the review methods were established prior to the review, and did the report justify any significant deviations from the protocol?	Yes	Yes	Yes	Yes	Yes	Yes	Yes	No	Yes	No	Yes	Yes	Yes	Yes	Yes
3. Did the review authors explain their selection of the study designs for inclusion in the review?	Yes	Yes	Yes	Yes	Yes	Yes	Yes	Yes	Yes	Yes	Yes	Yes	Yes	Yes	Yes
4. Did the review authors use a comprehensive literature search strategy?	Yes	Yes	Yes	No	Yes	Yes	Yes	No	Yes	Yes	Yes	Yes	Yes	Yes	Yes
5. Did the review authors perform study selection in duplicate?	No	Yes	Yes	Yes	Yes	Yes	Yes	Yes	Yes	Yes	Yes	Yes	Yes	Yes	Yes
6. Did the review authors perform data extraction in duplicate?	No	Yes	Yes	Yes	Yes	Yes	Yes	Yes	Yes	Yes	Yes	Yes	Yes	Yes	Yes
7. Did the review authors provide a list of excluded studies and justify the exclusions?	Yes	Yes	Yes	Yes	Yes	Yes	Yes	Yes	Yes	Yes	Yes	Yes	Yes	Yes	Yes
8. Did the review authors describe the included studies in adequate detail?	Yes	Yes	Yes	Yes	Yes	Yes	Yes	Yes	Yes	Yes	Yes	Yes	Yes	Yes	Yes
9. Did the review authors use a satisfactory technique for assessing the risk of bias (RoB) in individual studies included in the review?	No	Yes	Yes	Yes	Yes	Yes	Yes	No	Yes	No	Yes	Yes	Yes	Yes	Yes
10. Did the review authors report on the funding sources for the studies included in the review?	No	No	No	No	No	No	Yes	No	No	No	No	No	No	No	No
11. If meta-analysis was performed, did the review authors use appropriate methods for the statistical combination of results?	No	Yes	Yes	Yes	Yes	No	Yes	No	Yes	No	Yes	Yes	Yes	Yes	No
12. If meta-analysis was performed, did the review authors assess the potential impact of RoB in individual studies on the results of the meta-analysis or other evidence synthesis?	No	Yes	Yes	Yes	Yes	No	Yes	No	Yes	No	Yes	Yes	Yes	Yes	No
13. Did the review authors account for RoB in individual studies when interpreting/discussing the results of the review?	No	Yes	Yes	Yes	Yes	No	Yes	No	Yes	No	Yes	Yes	Yes	Yes	Yes
14. Did the review authors provide a satisfactory explanation for and discussion of any heterogeneity observed in the review results?	Yes	Yes	Yes	Yes	Yes	No	Yes	No	Yes	No	Yes	Yes	No	Yes	No
15. If they performed quantitative synthesis, did the review authors carry out an adequate investigation of publication bias (small study bias) and discuss its likely impact on the results of the review?	No	Yes	Yes	Yes	Yes	No	Yes	No	Yes	No	Yes	Yes	Yes	Yes	No
16. Did the review authors report any potential sources of conflict of interest, including any funding they received for conducting the review?	Yes	No	Yes	Yes	Yes	Yes	Yes	Yes	No	Yes	Yes	Yes	No	Yes	Yes
Total score	8	14	15	14	15	10	16	7	14	8	15	15	13	15	11
Methodological quality rating	Critically low	Moderate	High	Moderate	High	Low	High	Critically low	Moderate	Critically low	High	High	Moderate	High	Low

### 3.3 Certainty of evidence

In the certainty of the evidence, it was reported that for the balance variable in the Berg Balance Scale (BBS) and TUG tests, there was a moderate certainty of evidence (18, 26–28, 37, 41, 42), similar to the 30-s chair stand test (27, 37, 41, 42). While in the cardiorespiratory fitness variable through the 6-min walk test (18, 41), a high certainty of evidence was reported. On the contrary, in the direct muscle strength variables through handgrip strength (HGS) (41, 43) and knee extension (41, 43) tests, a very low certainty of the evidence was reported. The certainty of evidence is shown in Table 3; this allows us to recommend using active exergames in variables of balance and muscle strength, such as BBS, TUG, and 30-s chair stand tests in older people.

**Table 3:** GRADE assessment for the certainty of evidence.

Outcome	Review design	Risk of bias in individual review	Risk of publication bias	Inconsistency	Indirectness	Imprecision	Certainty of evidence
Balance							
BBS	4 RCT and 1 RCT/NRCT with 82 studies	Moderate	Not assessed	Moderate	Low risk	Moderate	Moderate
TUG	4 RCT and 1 RCT/NRCT with 98 studies	Moderate	Not assessed	Moderate	Low risk	Moderate	Moderate
Cardiorespiratory fitness							
6-min walk test	2 RCT with 40 studies	low	Not assessed	Moderate	Low risk	Moderate	High
Upper limb muscle strength							
HGS	1 RCT and 1 NRCT with 69 studies	High	Not assessed	High	Moderate	High	Very low
Lower limb muscle strength							
Knee extension test	1 RCT and 1 NRCT with 69 studies	High	Not assessed	High	Moderate	High	Very low
30-s chair stand test	3 RCT and 1 NRCT with 66 studies	Moderate	Not assessed	Moderate	Low risk	Moderate	Moderate

BBS, berg balance scale; TUG, timed up and go; HGS, handgrip strength; RCT, randomized controlled trial; NRCT, non-randomized controlled trial.

### 3.4 Studies characteristics

The characteristics of systematic reviews and meta-analyses analyzed in the overview indicated that 12 systematic reviews and meta-analyses were randomized controlled trials (17, 18, 25, 26, 28, 37–42, 44) and three systematic reviews and meta-analysis were non-randomized controlled trials (19, 27, 43). A total of 290 studies were analyzed in the selected systematic reviews and meta-analysis, totaling 15,832 participants with a mean age of 75.2 years (17–19, 25–28, 37–44). In general, older people who participated in the active exergames were apparently healthy (17, 18, 25–28, 37, 39, 41), pre-frail, and frail (19, 40, 44), which was determined by bone mineral densitometry and functional physical performance (19, 40, 44) and who participated in active exergames through the system with Xbox Kinect 360 (18, 19, 25, 26, 37, 38, 43), Nintendo Wii (17–19, 25, 27, 28, 37–44), and Play Station Movie (17, 18). The reviews analyzed report active control groups, specifically those that participated in traditional physical activity (17–19, 25, 26, 28, 37–42, 44), inactive control groups (no physical activity) (27, 28, 38, 41, 43), or who killed the basic activities of daily living (17, 18, 25, 41). More information about this topic is presented in Table 4.

**Table 4:** Systematic reviews and meta-analysis reporting outcomes in active exergames on physical performance.

References	Study design (SR and/or MA)	Baseline health status of the sample	Number of studies and participants in the intervention and control groups	The mean age of the sample	Activities performed in the consoles and active exergames and control groups	Training volume (total duration, weekly frequency, and time per game)	Physical performance data collection instruments	Main outcomes of the systematic reviews and meta-analysis	Quality assessment	Meta-analysis	PRISMA score
Chen et al. (26)	RCT	Older people, apparently healthy	20 (795; EG: 423; CG: 372).	EG: age 73.2 ± 5.3 years; CG: 73.8 ± 5.4 years.	EG: Xbox 360 Kinect. CG: Physical therapy exercises.	6/20 wk.; 2/3 wk. of 30/90 min.	BBS; FES-1 and Icon-FES	BBS↔; FES-1↑; Icon-FES↑ 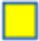	The Cochrane risk-of-bias tool	Yes	27
Fang et al. (37)	RCT	Older people, apparently healthy	16 (632; EG: 318; CG: 318).	EG: age 72.8 ± 7.0 years; CG: age 72.1 ± 6.5 years.	EG: Nintendo Wii and Xbox 360 Kinect. CG: traditional physical exercise or daily activities.	6/15 wk.; 2/3 wk. of 30/50 min.	TUG and 30-s chair stand	TUG↓; 30-s chair stand↑ 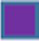	PEDro	Yes	25
Corregidor-Sánchez et al. (17)	RCT	Older people, apparently healthy	14 (Both groups: 491).	Both groups: age 77.2 ± 6.5 years.	EG: Nintendo Wii and Play Station movie. CG: traditional physical exercise or daily activities.	2/24 wk.; 1/5 wk.; no reported min.	FGA and GST.	FGA↔; GST↑ 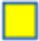 .	Not reported	Yes	27
Janhunen et al. (38)	RCT	Older people, apparently healthy	58 (Both groups: 3.774)	Both groups: age 74.3 ± 6.8 years	EG: Nintendo Wii and Xbox 360 Kinect. CG: traditional physical exercise or daily activities.	2/26 wk.; 2/3 wk. of 11/90 min.	FGA↑	FGA↑ 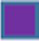	The Cochrane Risk of Bias 2 tool	Yes	26
Laufer et al. (39)	RCT	Older people, apparently healthy	7 (285; EG: 126; CG:159).	EG: age 71.5 ± 26.3 years; CG: age 76 ± 21.9 years.	EG: Nintendo Wii. CG: traditional physical exercise or daily activities.	6/20 wk.; 1/3 wk. of 30/60 min.	TUG	TUG↓ 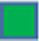	PEDro	No	13
Taylor et al. (42)	RCT	Older people, apparently healthy	18 (1.263; EG: 704; GC: 559).	EG: age 78.3 ± 10; GC: age 75 ± 8 years.	EG: Nintendo Wii. CG: traditional physical exercise or no intervention	3/20 wk.; 2/3 wk. of 40 min.	BBS	BBS↑ 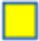	The Cochrane collaboration’s risk of bias tool	Yes	22
Pacheco et al. (28)	RCT	Older people, apparently healthy	12 (1.423; EG: 704; GC: 719).	EG: age 76 ± 6 years; 719 GC: age 76 ± 5 years.	EG: Nintendo Wii. CG: traditional physical exercise or no intervention	4/16 wk.; 2/3 wk. of 30/70 min.	BBS and TUG	BBS↑; TUG↑ 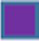	The Cochrane Handbook for Systematic reviews of interventions	Yes	26
Suleiman-Martos et al. (41)	RCT	Older people, apparently healthy	22 (Both groups: 1.208).	Both groups: age 74.9 ± 6.1 years.	EG: Nintendo Wii and Xbox 360 Kinect. CG: traditional physical exercise or no intervention.	3/24 wk.; 2/3 wk. of 15/120 min.	BBS; TUG; 6WMT; HGS; Knee extension and 30-s chair stand.	BBS↑; TUG↓; 6MWT↑; HGS↑; Knee extension ↔ and 30-s chair stand↑ 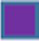 .	The Cochrane risk-of-bias tool	Yes	25
Zheng et al. (44)	RCT	Older people; frail and pre-frail.	7 (243; EG: 171; CG: 72).	EG: age 80.0 ± 6.2 years; CG: age 83.1 ± 5.0 years.	EG: Nintendo Wii and Xbox 360 Kinect. CG: traditional physical exercise or daily activities.	2/15 wk.; 2/3 wk. of 20/50 min.	FE	FES↔ 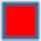	The Cochrane Collaboration’s tool for assessing the risk of bias	No	21
Afridi et al. (25)	RCT	Older people, apparently healthy	10 (Both groups: 442).	Both groups: age 65.8 ± 4.9 years.	EG: Nintendo Wii and Xbox 360 Kinect. CG: traditional physical exercise or daily activities.	8/12 wk.; 2/3 wk. of 30/60 min.	Tinetti’s falls efficacy scale and FES	Tinetti’s falls efficacy scale↔; FES↑ 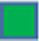	Not reported	No	12
Corregidor-Sánchez et al. (18)	RCT	Older people, apparently healthy	18 (Both groups: 772).	Both groups: age 71.1 ± 6.8 years.	EG: Nintendo Wii, Play Station movie, and Xbox 360 Kinect. CG: traditional physical exercise or daily activities.	2/24 wk.; 2/3 wk. of 20/50 min.	TUG and 6WMT	TUG↓; 6WMT↔ 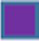	The Cochrane Collaboration’s tool for assessing the risk of bias	Yes	25
Ge et al. (19).	RCT/NRCT	Older people; frail and pre-frail.	23 (2.071; EG: 1.044; GC: 1.027).	EG: age 75 ± 5.5 years; CG: age 74.8 ± 5.1 years.	EG: Nintendo Wii and Xbox 360 Kinect. CG: traditional physical exercise or daily activities.	4/12 wk.; 1/3 wk. of 60 min.	FES, Icon-FES and Short FES-1	FES↑; Icon-FES↑; Short FES-1↑ 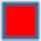	PEDro	No	17
Liu et al. (27)	RCT/NRCT	Older people, apparently healthy	10 (Both groups: 379).	Both groups: age 73.8 ± 6.2 years.	EG: Nintendo Wii. CG: No intervention	3/10 wk.; 2/3 wk. of 30/60 min.	BBS; TUG and 30-s chair stand.	BBS↑; TUG↓; 30-s chair stand↑ 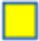 .	PEDro	Yes	23
Viana et al. (43)	RCT/NRCT	Older people; apparently healthy.	47 (Both groups: 1.760).	Both groups: age 84.1 ± 7.6 years.	EG: Nintendo Wii and Xbox 360 Kinect. CG: traditional physical exercise.	6/14 wk.; 1/5 wk. of 30/60 min.	HGS and knee extensión.	HGS↑; Knee extensión↑ 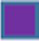 .	PEDro	Yes	25
Molina et al. (40)	RCT	Older people; frail and pre-frail.	8 (Both groups: 294).	Both groups: age 77.3 ± 4.4 years.	EG: Nintendo Wii. CG: traditional physical exercise or daily activities.	3/20 wk.; 1/5 wk. of 25/60 min.	BBS; TUG; ABC and LOB.	BBS↑; TUG↓; ABC↑ 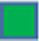 .	PEDro	No	19

PRISMA, Preferred Reporting Items for Systematic Reviews and Meta-analysis; SR, systematic review; MA, Meta-analysis; RCT, randomized controlled trial; NRCT, non-randomized controlled trial; EG, experimental groups; CG, control group; wk, week; Min, minutes; BBS, balance Berg scale; TUG, timed up and go test; HGS, handgrip strength; 6MWT, 6-min walk test; FGA, functional Gait assessment; GS, gait speed test; FES, falls efficacy scale; FES-1, falls efficacy scale-international; Icon-FES, Iconographical Falls Efficacy Scale; Short FES-1, short falls efficacy scale-international; ABC Scale, The Activities-specific Balance; 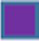, high methodological quality; 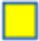, moderate methodological quality; 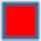, low methodological quality; 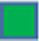
very low methodological quality; ↑, significant improvement; ↓ significant decrease; ↔, no significant difference.

### 3.5 Active exergames dosage

The dosage of the interventions with active exergames was diverse, ranging from 2 to 26 weeks with a frequency of one to five sessions per week with a duration of 30–60 min per session with moderate-to-vigorous intensities ranging from three to six in the 10-point rating of perceived exertion (RPE) (17–19, 25–28, 37–44).

### 3.6 Outcomes of overview

The results of systematic reviews and meta-analyses were synthesized. First, we qualitatively summarized the results of the systematic reviews and meta-analysis that reported the individual effects of different types of active exergames on physical performance in older people, then extracted data from the meta-analysis to better explain the effects of active exergames. Only 10 systematic reviews and meta-analyses qualified for meta-analysis that presented moderate and high methodological qualities.

### 3.7 Fall risk

Only four systematic reviews reported the fall risk measured through Tinetti’s falls efficacy scale (25), falls efficacy scale (19, 25, 44), short falls efficacy scale-international (19), efficacy scale-international, and iconographical fall efficacy scale (26). The individual results from systematic reviews indicate significant improvements in the groups with active exergames. However, conducting a meta-analysis was impossible due to the diversity of instruments and the small number of systematic reviews with meta-analysis; these findings cannot be confirmed.

### 3.8 Walking speed

Two reviews reported walking speed measures assessed through functional gait assessment (17, 38) and gait speed test (17). The individual results from systematic reviews indicate significant improvements in the groups with active exergames. However, conducting a meta-analysis was impossible due to the diversity of instruments and the small number of systematic reviews with meta-analysis; these findings cannot be confirmed.

### 3.9 Berg balance scale

**Figure 2:**
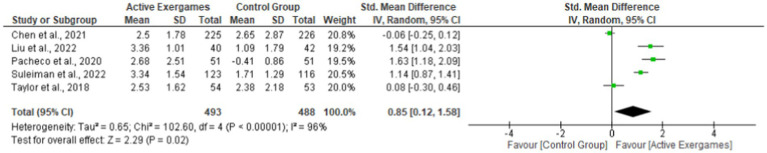
Effect of active exergames compared to control groups on the following outcome: Berg Balance Scale. The squares indicate the study-specific effect estimate. Bars indicate the width of the corresponding 95% confidence interval. The diamond centered on the summary effect estimate and the width indicate the corresponding 95% confidence interval.

Static balance was measured through the Berg Balance Scale (BBS) score. Five systematic reviews with meta-analysis analyzed the effect of active exergames on this measure (26–28, 41, 42), with 493 participants in the active exergames groups and 488 participants in the control groups. The results of the meta-analysis suggested that the active exergames significantly improved BBS score compared to control groups (SMD = 0.85; 95% CI = 0.12–1.58; I^2^ = 96%; *p* = 0.02), with high-level heterogeneity and using a fixed-effects model (Figure 2).

### 3.10 Timed up-and-go test

**Figure 3:**
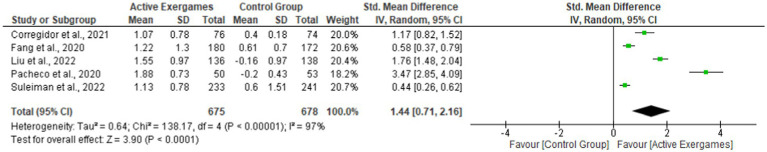
Effect of active exergames compared to control groups on the outcome: Timed up-and-go. The squares indicate the study-specific effect estimate. Bars indicate the width of the corresponding 95% confidence interval. The diamond centered on the summary effect estimate and the width indicate the corresponding 95% confidence interval.

Dynamic balance was measured using TUG. Five systematic reviews with meta-analysis were pooled for meta-analysis (18, 27, 28, 37, 41), with 675 participants in the active exergames groups and 678 participants in the control groups. Results indicate that active exergames significantly reduced time in TUG compared to control groups (SMD = 1.44; 95% CI = 0.71–2.16; I^2^ = 97%; *p* < 0.0001), with high-level heterogeneity using a fixed-effects model (Figure 3).

### 3.11 6-min walk test

**Figure 4:**
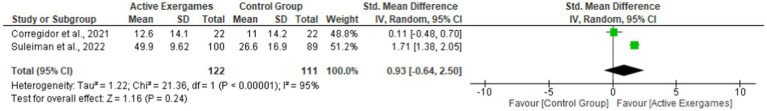
Effect of active exergames compared to control groups on 6-min walk test. The squares indicate the study-specific effect estimate. Bars indicate the width of the corresponding 95% confidence interval. The diamond centered on the summary effect estimate and the width indicate the corresponding 95% confidence interval.

Cardiorespiratory fitness was measured through the 6-min walk test. Two systematic reviews with meta-analysis were detected for meta-analysis (18, 41), with 122 participants in the active exergames groups and 111 participants in the control groups. The results indicate that the active exergames did not significantly differ in the 6-min walk test when compared to control groups (SMD = 0.93; 95% CI = −0.64 to 2.50; I^2^ = 95%; *p* = 0.24), with high-level heterogeneity using a fixed-effects model (Figure 4).

### 3.12 Handgrip strength

**Figure 5:**
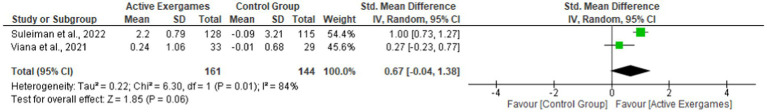
Effect of active exergames in comparison to the control group on handgrip strength. The squares indicate the study-specific effect estimate. Bars indicate the width of the corresponding 95% confidence interval. The diamond centered on the summary effect estimate and the width indicate the corresponding 95% confidence interval.

The HGS test measured maximal upper limb muscle strength. Two systematic reviews with meta-analysis were detected for meta-analysis (41, 43), with 161 participants in the active exergames groups and 144 participants in the control groups. The results indicate that the active exergames did not significantly differ in HGS when compared to control groups (SMD = 0.67; 95% CI = −0.04 to 1.38; I^2^ = 84%; *p* = 0.06), with high-level heterogeneity using a fixed-effects model (Figure 5).

### 3.13 30-s chair stand

**Figure 6:**
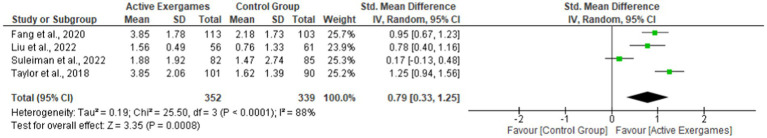
Effect of active exergames compared to the control group on the following outcome: 30-s chair stand. The squares indicate the study-specific effect estimate. Bars indicate the width of the corresponding 95% confidence interval. The diamond centered on the summary effect estimate and the width indicate the corresponding 95% confidence interval.

The 30-s chair stand test measured lower limb muscle strength. Four systematic reviews with meta-analysis were detected for meta-analysis (27, 37, 41, 42), with 352 participants in the active exergames groups and 339 participants in the control groups. Results indicate that the active exergames significantly improved in the 30-s chair stand test concerning control groups (SMD = 0.79; 95% CI = 0.33–1.25; I^2^ = 88%; *p* = 0.0008), with high-level heterogeneity using a fixed-effects model (Figure 6).

### 3.14 Knee extension

**Figure 7:**
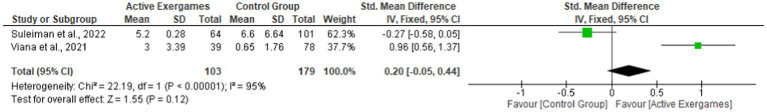
Effect of active exergames compared to the control group on the following outcome: knee extension. The squares indicate the study-specific effect estimate. Bars indicate the width of the corresponding 95% confidence interval. The diamond centered on the summary effect estimate and the width indicate the corresponding 95% confidence interval.

Two systematic reviews with meta-analysis were detected for the meta-analysis (41, 43), with 103 participants in the active exergames groups and 179 participants in the control groups. The results indicate that the active exergames, when compared to the control groups, did not significantly differ in knee extension (SMD = 0.20; 95% CI = −0.05 to 0.44; I^2^ = 95%; *p* = 0.12), with high-level heterogeneity using a fixed effects model (Figure 7).

**Figure 8:**
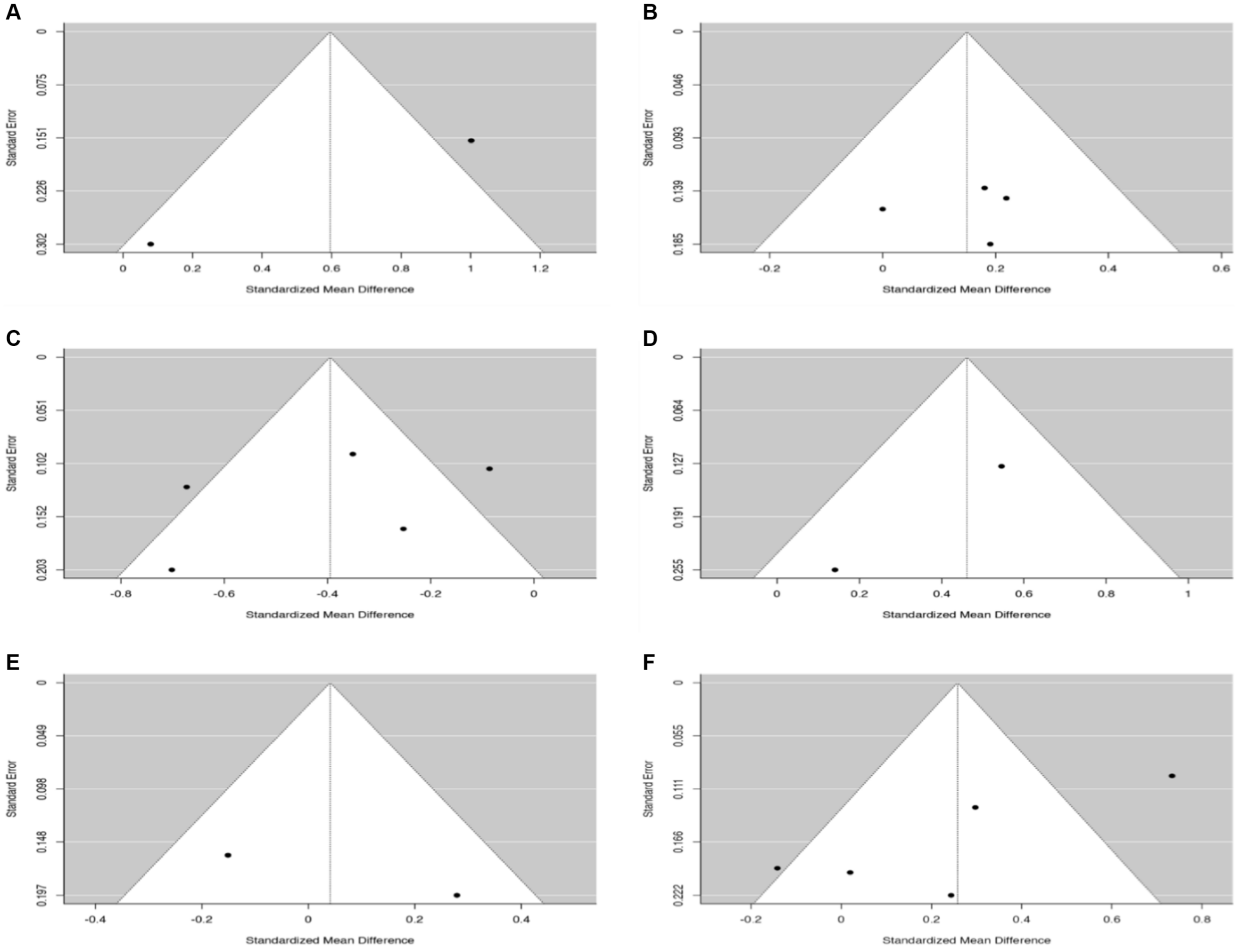
Egger’s test for assessment of potential publication bias. **(A)** 6-min walk test. **(B)** 30-s chair stand. **(C)** Timed up-and-go test. **(D)** Handgrip strength. **(E)** Knee extension. **(F)** Berg Balance Scale.

Egger’s regression asymmetry test observed no significant publication bias in the 30-s chair stand (*p* = 0.90) and in the TUG (*p* = 0.36). However, significant publication bias was observed in the following tests: 6-min walk (*p* = 0.001), HGS (*p* = 0.04), knee extension (*p* = 0.01), and BBS (*p* = 0.00). These results are presented in the funnel plot in Figure 8.

### 3.15 Adverse events

Only two systematic reviews and meta-analyses reported adverse events (25, 42). Specifically, the systematic review of Taylor et al. (42) reported minor musculoskeletal injuries (musculoskeletal strain) and vertigo sensation in both active exergames and control groups. For his part, Afridi et al. (25) reported that dropouts were due to back pain in some interventions with active exergames without requiring medical attention.

## 4 Discussion

This overview aimed to assess published peer-reviewed systematic reviews and meta-analyses concerning the effects of active exergames compared to active/passive controls on physical performance outcomes in older people. Fifteen systematic reviews and meta-analyses were identified, of which 10 provided sufficient information for meta-analysis and demonstrated moderate-to-high methodological quality. The main findings of the meta-analysis revealed statistically significant improvements in the BBS, TUG, and 30-s chair stand tests among the active exergames intervention groups compared to the control groups. However, no significant differences were found in the 6-min walk, HGS, and knee extension tests when comparing the two groups.

### 4.1 Balance

In the present overview, significant differences were detected in favor of the active exergames groups in static balance measured through the BBS (SMD = 0.85; 95% CI = 0.12–1.58; I^2^ = 96%; *p* = 0.02) concerning the control groups. This is similar to that reported in a meta-analysis by Lesinski et al. (45), which shows statistically significant improvements (*p* = 0.03) in BBS in favor of interventions in older people through balance training concerning active/inactive control groups. Similarly, Lemos et al. (46), in a meta-analysis of older people, showed statistically significant improvements (*p* = 0.010) in BBS in favor of multicomponent training interventions concerning active/inactive control groups. Aging leads to alterations in the vestibular, sensory, and proprioceptive systems that lead to decreased balance, which increases the fall risk in older people (47). In active exergames interventions, actions involving visual, auditory, and proprioceptive feedback movements are performed, which would favor the balance of older people; in the same way, to advance in the game phases, the difficulty increases; this implies a greater complexity in the movements to be executed and the intensity of the game (48). Therefore, early detection of balance changes or alterations is significant. In this sense, the BBS is a simple indirect test, easy to apply, with widespread use, low cost, and high reliability (0.96–0.98) and validity (0.97–0.99) to assess older people (49).

Regarding dynamic balance, the meta-analysis reported significant differences in favor of the active exergames groups for control groups measured through the TUG (SMD = 1.44; 95% CI = 0.71–2.16; I^2^ = 97%; *p* < 0.0001). Similarly, interventions using elastic bands in older people have shown statistically significant improvements (*p* < 0.01) in TUG compared to active/inactive control groups in previous meta-analyses performed (50, 51). In the same way, Labata-Lezaun et al. (7), in a meta-analysis of older people, showed statistically significant improvements in TUG (*p* = 0.0001) in favor of multicomponent training interventions regarding active/inactive control groups. However, interventions using active exergames in a shorter period range from 3 to 20 weeks with 2–3 sessions per week for 30–60 min, compared to interventions using elastic bands, which range from 8 to 28 weeks with 1–3 sessions per week for 30–90 min (51), and multicomponent training, which range from 9 to 48 weeks with 2–5 sessions for 30–90 min (7); this can lead to an increase in lower limb muscle strength along with balance related to improvements in BBS and TUG; these adaptations can reduce the risk of fear and falls in older people (22). Limitations in lower limb mobility, such as alterations in dynamic balance, actions such as walking, getting up from a chair, or both, are indicators of impaired functional independence (22). The TUG has demonstrated high reliability (0.98) and validity (0.98) for measuring dynamic balance indirectly in older people (52).

The previously mentioned measurements to assess balance (BBS and TUG) present high reliability, validity, and wide use in the scientific literature related to older people (49, 52). On the other hand, direct methods such as oscillography have higher reliability and validity values (0.99) and provide values of the center of pressure (53). However, having oscillography equipment is more expensive, and it is difficult for some care centers for older people (residences, neighborhood councils, and groups of older people) to have access to this instrumentation, while health and sports science professionals cannot always move this material to the spaces where older people practice physical activity (53). Therefore, having evidence of the benefits of active exergames on balance, even if measured indirectly, provides relevant information for its use in the clinical and health context (22, 54, 55).

### 4.2 Cardiorespiratory fitness

In the present overview, no significant differences in cardiorespiratory fitness measured by the 6-min walk test were reported in the active exergames group compared to control groups (SMD = 0.93; 95% CI = −0.64 to 2.50; I^2^ = 95%; *p* = 0.24). In contrast to active exergames, a meta-analysis in older people by Stern et al. (56) reported statistically significant improvements (*p* = 0.01) in the 6-min walk test in favor of high-intensity interval training (HIIT) interventions concerning active/inactive control groups. Similar to that reported by Labata-Lezaun et al. (7) in a meta-analysis, statistically significant improvements (*p* = 0.01) were detected in favor of multicomponent training interventions concerning active/inactive control groups in 6-min walk test in older people; this may be because active exergames interventions are performed at a low-to-moderate intensity during most games, in the first weeks of neuromuscular adaptation to the intervention, and along with short minutes performed during the game stage that cannot be individualized for each person (57). During aging, neuromuscular deterioration occurs, accompanied by a decrease in cardiorespiratory fitness associated with decreased mobility, which can affect independence in older people (58). The 6-min walk test is an indirect method to measure cardiorespiratory fitness in older people, unlike ergospirometry, which assesses cardiorespiratory fitness directly in older people by maximum oxygen consumption, showing a high reliability and validity of 0.95 (59). However, this is a method of high economic cost and not easily accessible compared to the 6-min walk test, which has proven to be a simple, easy, and quick application test with a high reliability and validity of 0.96 that measures cardiorespiratory fitness in older people (60).

### 4.3 Upper limb muscle strength

In the present overview, no significant differences were detected in maximal upper limb muscle strength measured by HGS between active exergames and control groups. Similar to that reported by Daryanti Saragih et al. (61) in a meta-analysis, no statistically significant improvements (*p* = 0.40) in HGS were observed in interventions using elastic band training concerning active/inactive control groups in older people. However, a meta-analysis in older people by Khalafi et al. (62) reported statistically significant improvements (*p* = 0.001) in HGS in concurrent training interventions to active/inactive control groups. Interventions using active exergames, unlike strategies using traditional physical activity (i.e., concurrent training), do not produce an increase in the load used because only bodyweight movements are executed, which may lead to less neuromuscular activation, muscle hypertrophy, strength, and muscle quality in wrist flexor-extensor muscles (41, 43, 63, 64); this may be because interventions using Xbox Kinect do not manipulate any control in hand hence do not grasp or hold any control, because the sensor that tracks the movements executed in the game is in a camera that is in front of the player (65).

In contrast to the Nintendo Wii, the movements to perform the games are executed by a controller that must be grasped and held by hand because the sensor is on the controller (65). In neither of the two interventions using active exergames is there an improvement in HGS because there is no stimulus that leads to an increase in the load used in the upper limbs, mainly in the wrist flexor-extensor muscles that leads to an increase in HGS (65). The HGS is an effective direct method for assessing upper limb muscle strength of the wrist flexor-extensor muscles in older people, with a high reliability and validity of 0.98 (66). A good performance on this test is considered a good predictor of a lower risk of all-cause mortality (67).

### 4.4 Lower limb muscle strength

Similarly, the overview did not detect significant differences in lower limb muscle strength measured by the knee extension test between the active exergames groups compared to the control groups. However, a meta-analysis by Khalafi et al. (62) reported statistically significant improvements (*p* = 0.001) in favor of multicomponent training interventions concerning active/inactive control groups in older people. Neural adaptations and/or alterations at the muscle fiber level or changes in the main components of excitation-contraction coupling may explain strength gains in traditional physical activities such as resistance training (43). However, the lack of overload in active exergames interventions may have limited the significant isometric strength gains for the lower limbs in the knee extension test (43). The knee extension test is a direct method that measures maximal isometric strength in the knee extensor muscles in older people with a high reliability and validity of 0.98 (68).

Another result reported in the present overview had significant differences in favor of the active exergames groups in the 30-s chair stand test (SMD = 0.79; 95% CI = 0.33–1.25; I^2^ = 88%; *p* = 0.0008) compared to control groups. Similar to that reported by Labata-Lezaun et al. (7) in a meta-analysis, statistically significant improvements (*p* = 0.002) in the 30-s chair stand test were observed in interventions through multicomponent training regarding active/inactive control groups in older people. Similarly, de Oliveira et al. (69), in a meta-analysis, reported statistically significant improvements in the 30-s chair stand test in favor of interventions using elastic band training concerning active/inactive control groups in older people. Some games that are performed through interventions with active exergames such as bowling, table tennis, athletics, volleyball, soccer, Kinect adventure, and Wii fit participants adopt a knee angle at 90°, similar to the angle considered for the realization of the 30-s chair stand test, which can generate adaptations at the neuromuscular level that can lead to improvements in this test (70). Getting up from a chair independently is essential for safe performance in activities of daily living (71). Community-dwelling older people with and without health problems stand and sit from a chair between 33 and 71 times daily (72). The 30-s chair stand test measures lower limb muscle strength indirectly by the number of repetitions a person can execute by standing and sitting from a chair for 30 s (71), with high reliability and validity of 0.97 for measuring older people (73).

### 4.5 Methodological quality and certainty of evidence

The AMSTAR-2 program has been used to measure and evaluate the methodological quality, while certainty of evidence was obtained using GRADE (30, 74). Importantly, it allows us to deliver conclusive information on the variables analyzed (30, 74). A review by El-Kotob et al. (75) on the effect of resistance training in adults showed a low methodological quality with AMSTAR-2; this is similar to that reported by Leung et al. (76) in an abstract that analyzed the effect of Tai Chi training on indicators of functionality in older people showing low methodological quality with AMSTAR-2. However, in the present overview, a moderate (13–14 points)-to-high (15–16 points) methodological quality was reported in the 10 meta-analyses detected for the analysis (17, 18, 26–28, 37, 38, 41–43). In comparison, there was moderate-to-high certainty of evidence in the BBS, TUG, 30-s chair stand, and 6-min walk tests. Similar to the findings in the study by Shen et al. (77), which demonstrated moderate-to-high quality evidence on physical performance through resistance, balance, and aerobic training in older people through GRADE, this allows us to recommend interventions using active exergames to improve the BBS, TUG, and 30-s chair stand tests in apparently healthy older people.

### 4.6 Active exergames dosage

Regarding the dose used by the interventions with active exergames that report results for the BBS, TUG, and 30-s chair stand tests, a duration between 3 and 20 weeks was reported with 2–3 weekly sessions for 30–60 min, with moderate-to-vigorous intensities ranging from 3 to 6 in the 10-point RPE (78). Another type of training with elastic bands has shown statistically significant improvements on these variables with interventions ranging from 8 to 28 weeks with 1–3 sessions per week for 30–90 min with intensities of 25–80% of the one-repetition maximum (1RM) from 1 to 2 sets per session of 5–20 repetitions per set in upper and lower limbs exercises as reported in various meta-analyses in older people (50, 51, 61, 69). As the multicomponent training has shown statistically significant improvements in these previously mentioned variables (BBS and TUG), the following is a good example with interventions ranging from 9 to 48 weeks with 2 to 5 sessions per week for 30–90 min with upper and lower limbs exercises, as reported in different meta-analyses (7, 46). However, no significant differences were reported regarding active exergames in HGS and knee extension tests in the present overview. However, the 30-s chair stand test was significant. In other types of interventions, such as concurrent training with a duration of 4 weeks to 12 months with 3–5 sessions per week for 30–140 min with intensities of 60–90% of 1RM and 60–90% of maximum heart rate (HR_max_) with upper and lower limbs exercises, statistically significant improvements have been reported in HGS and knee extension tests as demonstrated in a meta-analysis in older people (62). However, HIIT training has demonstrated statistically significant improvements in the 6-min walk test with interventions of 6–28 weeks of 2–7 sessions per week for 30–60 min with intervals of 6–30 at intensities ≥90% of the HR_max_ as demonstrated in a meta-analysis in older people (56). However, these interventions (HIIT) have shown low adherence due to the high intensities at which the intervals are performed as the repetitive actions executed (79). In contrast, active exergames have shown high adherence to the interventions with high enjoyment of the activity performed due to the diversity of games that are executed during the interventions (80); therefore, it could serve as a complement to other physical activity strategies.

### 4.7 Strengths and limitations of the overview

The limitations of the present overview are as follows: (*i*) the low number of similar assessments among the systematic reviews and meta-analysis available to meta-analyze the data, limiting the recommendations for some assessments analyzed, and (*ii*) the high heterogeneity found in the group analyses may limit the recommendations of the results obtained; however, finding low heterogeneity in systematic reviews and meta-analyses is unlikely as well as the optimal size of information due to the diversity in the number of population found in the available studies (81–83). Afonso et al. (81) recommended being careful in misinterpreting the concept of publication bias in meta-analyses (i.e., using definitive rather than provisional statements), misusing funnel plots and associated statistical tests to assess potential publication bias, and misinterpreting subsequent results. The main strengths are as follows: (*i*) the use of 7 (PubMed, Web of Science, Scopus, CINAHL, Cochrane Library, MEDLINE, and Psychology and Behavioral Sciences) generic databases, which broadens the scope of the search; (*ii*) performing meta-analysis which allows quantifying what is reported in the systematic reviews and meta-analysis; (*iii*) comparison with active/inactive control groups which increases the quality in the comparator on its effect on the variables analyzed; (*iv*) meta-analyzing only systematic reviews with moderate-to-high methodological quality this allows recommending intervention on the variables analyzed; and (*v*) analyzing different consoles and virtual reality games that allow a broader vision of their impact on health status in older people. Future overviews could analyze systematic reviews on the effect of active exergames on psychoemotional and physiological variables or see if there are differences between the type of consoles or games on physical performance in older people.

### 4.8 Practical applications

This overview of systematic reviews with meta-analysis highlights the potential impact of active exergames to improve physical performance in the BBS, TUG, and 30-s chair stand tests in apparently healthy older people with interventions ranging from 3 to 20 weeks, the improvement in the indicated tests being related to greater functional independence and lower fall risk (18, 26–28, 37, 41, 42). Therefore, active exergames could serve as a complementary physical activity strategy in clinical practice and could be used in primary healthcare, community centers, nursing homes, and physical activity programs oriented to older people.

## 5 Conclusion

Interventions utilizing active exergames have shown significant improvements in the static and dynamic balance and lower limb muscle strength of apparently healthy older people, compared to control groups of active/inactive participants, as measured by the BBS, TUG, and 30-s chair stand tests. However, no significant differences were found in the 6-min walk, HGS, and knee extension tests. The certainty of the evidence was rated as moderate-to-high, thus suggesting that active exergames can be recommended as a physical activity strategy to enhance the performance of older people in terms of balance and lower limb muscle strength. Nevertheless, it is essential to supplement this intervention with activities focusing on improving cardiorespiratory fitness and upper limb muscle strength.

## Data availability statement

The datasets generated during and/or analyzed during the current research are available from the Corresponding author upon reasonable request.

## Author contributions

JH-M and PV-B: conceptualization, software, writing-original draft preparation. JH-M, CM-V, and PV-B: methodology. JH-M, CM-V, FR-E, and PV-B: formal analysis. JH-M, FR-E, CM-V, EG-M, TH-V, BB, MC-C, and PV-B: investigation and writing-review and editing. PV-B, EG-M, TH-V, and BB: supervision. All authors have read and agreed to the published version of the manuscript.

## References

[1] Değer TB Saraç ZF Savaş ES Akçiçek SF. The relationship of balance disorders with falling, the effect of health problems, and social life on postural balance in the elderly living in a district in Turkey. Geriatrics. (2019) 4:37. doi: 10.3390/geriatrics4020037 31108836 PMC6630729

[2] Freiberger E Sieber CC Kob R. Mobility in older community-dwelling persons: a narrative review. Front Physiol. (2020) 11:881. doi: 10.3389/fphys.2020.00881, PMID: 33041836 PMC7522521

[3] Khodadad Kashi S Mirzazadeh ZS Saatchian V. A systematic review and meta-analysis of resistance training on quality of life, depression, muscle strength, and functional exercise capacity in older adults aged 60 years or more. Biol Res Nurs. (2022) 25:88–106. doi: 10.1177/10998004221120945 35968662

[4] WHO. (2021). World Health Organization falls. Available at: https://www.who.int/news-room/fact-sheets/detail/falls

[5] Romero-Ortuño R Martínez-Velilla N Sutton R Ungar A Fedorowski A Galvin R . Network physiology in aging and frailty: the grand challenge of physiological reserve in older adults. Front Netw Physiol. (2021) 1:712430. doi: 10.3389/fnetp.2021.712430 36925570 PMC10012993

[6] Valdés-Badilla PA Gutiérrez-García C Pérez-Gutiérrez M Vargas-Vitoria R López-Fuenzalida A. Effects of physical activity governmental programs on health status in independent older adults: a systematic review. J Aging Phys Act. (2019) 27:265–75. doi: 10.1123/japa.2017-0396, PMID: 29989461

[7] Labata-Lezaun N González-Rueda V Llurda-Almuzara L López-De-Celis C Rodríguez-Sanz J Bosch J . Effectiveness of multicomponent training on physical performance in older adults: a systematic review and meta-analysis. Arch Gerontol Geriatr. (2023) 104:104838. doi: 10.1016/j.archger.2022.104838, PMID: 36272227

[8] Bull FC Al-Ansari SS Biddle S Borodulin K Buman MP Cardon G . World Health Organization 2020 guidelines on physical activity and sedentary behaviour. Br J Sports Med. (2020) 54:1451–62. doi: 10.1136/bjsports-2020-102955, PMID: 33239350 PMC7719906

[9] Fragala MS Cadore EL Dorgo S Izquierdo M Kraemer WJ Peterson MD . Resistance training for older adults: position statement from the national strength and conditioning association. J Strength Cond Res. (2019) 33:2019–52. doi: 10.1519/JSC.0000000000003230, PMID: 31343601

[10] Schöttl SE Schnitzer M Savoia L Kopp M. Physical activity behavior during and after Covid-19 stay-at-home orders-a longitudinal study in the Austrian, German, and Italian Alps. Front Public Health. (2022) 10:901763. doi: 10.3389/fpubh.2022.901763, PMID: 35712287 PMC9194442

[11] De Maio M Bratta C Iannaccone A Castellani L Foster C Cortis C . Home-based physical activity as a healthy aging booster before and during Covid-19 outbreak. Int J Environ Res Public Health. (2022) 19:317. doi: 10.3390/ijerph19074317 PMC8998434 35410000

[12] Buyle M Jung Y Pavlou M Gonzalez SC Bamiou D-E. The role of motivation factors in exergame interventions for fall prevention in older adults: a systematic review and meta-analysis. Front Neurol. (2022) 13:903673. doi: 10.3389/fneur.2022.903673 35989930 PMC9388774

[13] Ismail NA Hashim HA Ahmad Yusof H. Physical activity and exergames among older adults: a scoping review. Games Health J. (2022) 11:1–17. doi: 10.1089/g4h.2021.0104, PMID: 34851732

[14] Jinhui L Li L Huo P Ma C Wang L Theng YL. Wii or Kinect? A pilot study of the Exergame effects on older Adults' physical fitness and psychological perception. Int J Environ Res Public Health. (2021) 18:12939. doi: 10.3390/ijerph182412939 34948547 PMC8701390

[15] Jacobson G. (2021). When costs are a barrier to getting health care: reports from older adults in the United States and other high-income countries. Available at: https://www.commonwealthfund.org/publications/surveys/2021/oct/when-costs-are-barrier-getting-health-care-older-adults-survey

[16] National Health Service of the Kingdom United (2015). This article is more than 7 years old analysis ageing Britain: two-fifths of Nhs budget is spent on over-65s. Available at: https://www.theguardian.com/society/2016/feb/01/ageing-britain-two-fifths-nhs-budget-spent-over-65s

[17] Corregidor-Sánchez AI Segura-Fragoso A Rodríguez-Hernández M Criado-Alvarez JJ González-Gonzalez J Polonio-López B. Can exergames contribute to improving walking capacity in older adults? A systematic review and meta-analysis. Maturitas. (2020) 132:40–8. doi: 10.1016/j.maturitas.2019.12.006, PMID: 31883662

[18] Corregidor-Sánchez AI Segura-Fragoso A Rodríguez-Hernández M Jiménez-Rojas C Polonio-López B Criado-Álvarez JJ. Effectiveness of virtual reality technology on functional mobility of older adults: systematic review and meta-analysis. Age Ageing. (2021) 50:370–9. doi: 10.1093/ageing/afaa197, PMID: 33068106

[19] Ge L Su TT An Y Mejía ST. The effectiveness of exergames on fear of falling in community-dwelling older adults: a systematic review. Aging Ment Health. (2022) 26:1306–17. doi: 10.1080/13607863.2021.1950615, PMID: 34291684

[20] Fu AS Gao KL Tung AK Tsang WW Kwan MM. Effectiveness of exergaming training in reducing risk and incidence of falls in frail older adults with a history of falls. Arch Phys Med Rehabil. (2015) 96:2096–102. doi: 10.1016/j.apmr.2015.08.427, PMID: 26360975

[21] Yang C-M Chen Hsieh JS Chen Y-C Yang S-Y Lin H-CK. Effects of Kinect exergames on balance training among community older adults: a randomized controlled trial. Medicine. (2020) 99:e21228. doi: 10.1097/MD.0000000000021228, PMID: 32664177 PMC7360267

[22] Hernandez-Martínez J Ramirez-Campillo R Álvarez C Valdés-Badilla PA Moran J Izquierdo M. Effects of active exergames training on physical functional performance in older females (Efectos del entrenamiento de exergames activos sobre el rendimiento físico funcional en mujeres mayores). Cult Cien Dep. (2022) 17:77–84 doi: 10.12800/ccd.v17i51.1658

[23] Yu T-C Chiang C-H Wu P-T Wu W-L Chu IH. Effects of Exergames on physical fitness in middle-aged and older adults in Taiwan. Int J Environ Res Public Health. (2020) 17:2565. doi: 10.3390/ijerph17072565, PMID: 32276515 PMC7177712

[24] Liao YY Chen IH Hsu WC Tseng HY Wang RY. Effect of exergaming versus combined exercise on cognitive function and brain activation in frail older adults: A randomised controlled trial. Ann Phys Rehabil Med. (2021) 64:101492. doi: 10.1016/j.rehab.2021.101492, PMID: 33454398

[25] Afridi A Rathore FA Nazir SNB. Wii fit for balance training in elderly: a systematic review. J Coll Physicians Surg Pak. (2021) 30:559–66. doi: 10.29271/jcpsp.2021.05.559, PMID: 34027869

[26] Chen Y Zhang Y Guo Z Bao D Zhou J. Comparison between the effects of exergame intervention and traditional physical training on improving balance and fall prevention in healthy older adults: a systematic review and meta-analysis. J Neuroeng Rehabil. (2021) 18:164. doi: 10.1186/s12984-021-00917-0, PMID: 34819097 PMC8611920

[27] Liu H Xing Y Wu Y. Effect of Wii fit exercise with balance and lower limb muscle strength in older adults: a meta-analysis. Front Med. (2022) 9:812570. doi: 10.3389/fmed.2022.812570, PMID: 35602499 PMC9120538

[28] Pacheco TBF De Medeiros CSP De Oliveira VHB Vieira ER De Cavalcanti FAC. Effectiveness of exergames for improving mobility and balance in older adults: a systematic review and meta-analysis. Syst Rev. (2020) 9:163. doi: 10.1186/s13643-020-01421-7, PMID: 32682439 PMC7368979

[29] Page M Mckenzie JE Bossuyt PM Boutron I Hoffmann TC Mulrow CD . The Prisma 2020 statement: an updated guideline for reporting systematic reviews. BMJ. (2021) 372:n71. doi: 10.1136/bmj.n71 33782057 PMC8005924

[30] Shea BJ Reeves BC Wells G Thuku M Hamel C Moran J . Amstar 2: a critical appraisal tool for systematic reviews that include randomised or non-randomised studies of healthcare interventions, or both. BMJ. (2017) 358:j4008. doi: 10.1136/bmj.j4008 28935701 PMC5833365

[31] Guyatt G Oxman AD Akl EA Kunz R Vist G Brozek J . Grade guidelines: 1. Introduction-grade evidence profiles and summary of findings tables. J Clin Epidemiol. (2011) 64:383–94. doi: 10.1016/j.jclinepi.2010.04.026, PMID: 21195583

[32] Weir CJ Butcher I Assi V Lewis SC Murray GD Langhorne P . Dealing with missing standard deviation and mean values in meta-analysis of continuous outcomes: a systematic review. BMC Med Res Methodol. (2018) 18:25. doi: 10.1186/s12874-018-0483-0, PMID: 29514597 PMC5842611

[33] Stogiannis D Siannis F Androulakis E. Heterogeneity in meta-analysis: a comprehensive overview. Int J Biostat. (2023). doi: 10.1515/ijb-2022-0070 36961993

[34] Jiantong S Wenming F Yike W Qiyuan Z Billong Laura F Jingya L. Efficacy and safety of aliskiren combination therapy: a protocol for an umbrella review. BMJ Open. (2021) 11:e043807. doi: 10.1136/bmjopen-2020-043807 PMC7944987 33687953

[35] Li X Wei W Wang Y Wang Q Liu Z. Touching-bone acupuncture in the treatment of chronic pain: a protocol for an overview of systematic reviews and meta-analysis. Medicine. (2021) 100:e27195. doi: 10.1097/MD.0000000000027195, PMID: 34797271 PMC8601311

[36] Sterne JA Egger M Smith GD. Systematic reviews in health care: investigating and dealing with publication and other biases in meta-analysis. BMJ. (2001) 323:101–5. doi: 10.1136/bmj.323.7304.101, PMID: 11451790 PMC1120714

[37] Fang Q Ghanouni P Anderson SE Touchett H Shirley R Fang F . Effects of exergaming on balance of healthy older adults: a systematic review and meta-analysis of randomized controlled trials. Games Health J. (2020) 9:11–23. doi: 10.1089/g4h.2019.0016, PMID: 31800322

[38] Janhunen M Karner V Katajapuu N Niiranen O Immonen J Karvanen J . Effectiveness of Exergame intervention on walking in older adults: a systematic review and meta-analysis of randomized controlled trials. Phys Ther. (2021) 101:pzab152. doi: 10.1093/ptj/pzab152 34160022 PMC8459884

[39] Laufer Y Dar G Kodesh E. Does a Wii-based exercise program enhance balance control of independently functioning older adults? A systematic review. Clin Interv Aging. (2014) 9:1803–13. doi: 10.2147/CIA.S69673, PMID: 25364238 PMC4211857

[40] Molina KI Ricci NA De Moraes SA Perracini MR. Virtual reality using games for improving physical functioning in older adults: a systematic review. J Neuroeng Rehabil. (2014) 11:156. doi: 10.1186/1743-0003-11-156, PMID: 25399408 PMC4247561

[41] Suleiman-Martos N García-Lara R Albendín-García L Romero-Béjar JL Cañadas-De La Fuente GA Monsalve-Reyes C . Effects of active video games on physical function in independent community-dwelling older adults: a systematic review and meta-analysis. J Adv Nurs. (2022) 78:1228–44. doi: 10.1111/jan.15138, PMID: 34935178

[42] Taylor LM Kerse N Frakking T Maddison R. Active video games for improving physical performance measures in older people: a meta-analysis. J Geriatr Phys Ther. (2018) 41:108–23. doi: 10.1519/JPT.0000000000000078, PMID: 26974212 PMC5895114

[43] Viana RB De Oliveira VN Dankel SJ Loenneke JP Abe T Da Silva WF . The effects of exergames on muscle strength: a systematic review and meta-analysis. Scand J Med Sci Sports. (2021) 31:1592–611. doi: 10.1111/sms.13964, PMID: 33797115

[44] Zheng L Li G Wang X Yin H Jia Y Leng M . Effect of exergames on physical outcomes in frail elderly: a systematic review. Aging Clin Exp Res. (2020) 32:2187–200. doi: 10.1007/s40520-019-01344-x, PMID: 31520334

[45] Lesinski M Hortobágyi T Muehlbauer T Gollhofer A Granacher U. Effects of balance training on balance performance in healthy older adults: a systematic review and meta-analysis. Sports Med. (2015) 45:1721–38. doi: 10.1007/s40279-015-0375-y, PMID: 26325622 PMC4656699

[46] Lemos ECWM Guadagnin EC Mota CB. Influence of strength training and multicomponent training on the functionality of older adults: systematic review and meta-analysis. Rev Bras Cineantrop Desemp Hum. (2020) 22:1–20 doi: 10.1590/1980-0037.2020v22e6070

[47] Cuevas-Trisan R. Balance problems and fall risks in the elderly. Phys Med Rehabil Clin N Am. (2017) 28:727–37. doi: 10.1016/j.pmr.2017.06.006 29031339

[48] Cordeiro HIP De Mello Alves Rodrigues AC Alves MR Gatica-Rojas V Maillot P De Moraes Pimentel D . Exercise with active video game or strength/balance training? Case reports comparing postural balance of older women. Aging Clin Exp Res. (2020) 32:543–5. doi: 10.1007/s40520-019-01219-1 31124058

[49] Downs S Marquez J Chiarelli P. The Berg balance scale has high intra- and inter-rater reliability but absolute reliability varies across the scale: a systematic review. J Physiother. (2013) 59:93–9. doi: 10.1016/S1836-9553(13)70161-9, PMID: 23663794

[50] Efendi F Tonapa SI Has EMM Ho KHM. Effects of chair-based resistance band exercise on physical functioning, sleep quality, and depression of older adults in long-term care facilities: systematic review and meta-analysis. Int J Nurs Sci. (2023) 10:72–81. doi: 10.1016/j.ijnss.2022.12.002, PMID: 36860706 PMC9969069

[51] Yeun YR. Effectiveness of resistance exercise using elastic bands on flexibility and balance among the elderly people living in the community: a systematic review and meta-analysis. J Phys Ther Sci. (2017) 29:1695–9. doi: 10.1589/jpts.29.1695, PMID: 28932015 PMC5599848

[52] Aslankhani MA Farsi A Fathirezaie Z Zamani Sani SH Aghdasi MT. Validity and reliability of the timed up and go and the anterior functional reach tests in evaluating fall risk in the elderly. Salmand Iran J Ageing. (2015) 10:16–25. Available at: http://salmandj.uswr.ac.ir/article-1-720-en.html

[53] Li Z Liang YY Wang L Sheng J Ma SJ. Reliability and validity of center of pressure measures for balance assessment in older adults. J Phys Ther Sci. (2016) 28:1364–7. doi: 10.1589/jpts.28.1364, PMID: 27190484 PMC4868244

[54] Karahan AY Tok F Taşkın H Kuçuksaraç S Başaran A Yıldırım P. Effects of Exergames on balance, functional mobility, and quality of life of geriatrics versus home exercise programme: randomized controlled study. Cent Eur J Public Health. (2015) 23:S14–8. doi: 10.21101/cejph.a4081, PMID: 26849537

[55] Padala KP Padala PR Lensing SY Dennis RA Bopp MM Parkes CM . Efficacy of Wii-fit on static and dynamic balance in community dwelling older veterans: a randomized controlled pilot trial. J Aging Res. (2017) 2017:4653635. doi: 10.1155/2017/4653635 28261500 PMC5316445

[56] Stern G Psycharakis SG Phillips SM. Effect of high-intensity interval training on functional movement in older adults: a systematic review and meta-analysis. Sports Med Open. (2023) 9:5. doi: 10.1186/s40798-023-00551-1, PMID: 36641767 PMC9840985

[57] Berg J Haugen G Wang AI Moholdt T. High-intensity exergaming for improved cardiorespiratory fitness: a randomised, controlled trial. Eur J Sport Sci. (2022) 22:867–76. doi: 10.1080/17461391.2021.1921852 33944698

[58] Gabrielle Dupuy E Besnier F Gagnon C Breton J Vincent T Grégoire C-A . Cardiorespiratory fitness moderates the age-related association between executive functioning and mobility: evidence from remote assessments. Innova Aging. (2022) 7:igac077. doi: 10.1093/geroni/igac077 PMC9950718 36846304

[59] Dougherty RJ Lindheimer JB Stegner AJ Van Riper S Okonkwo OC Cook DB. An objective method to accurately measure cardiorespiratory fitness in older adults who cannot satisfy widely used oxygen consumption criteria. J Alzheimers Dis. (2018) 61:601–11. doi: 10.3233/JAD-170576, PMID: 29226867 PMC5745283

[60] Guerra-Balic M Oviedo GR Javierre C Fortuño J Barnet-López S Niño O . Reliability and validity of the 6-min walk test in adults and seniors with intellectual disabilities. Res Dev Disabil. (2015) 47:144–53. doi: 10.1016/j.ridd.2015.09.011, PMID: 26426514

[61] Daryanti Saragih I Yang YP Saragih IS Batubara SO Lin CJ. Effects of resistance bands exercise for frail older adults: a systematic review and meta-analysis of randomised controlled studies. J Clin Nurs. (2022) 31:43–61. doi: 10.1111/jocn.15950, PMID: 34289511

[62] Khalafi M Sakhaei MH Rosenkranz SK Symonds ME. Impact of concurrent training versus aerobic or resistance training on cardiorespiratory fitness and muscular strength in middle-aged to older adults: a systematic review and meta-analysis. Physiol Behav. (2022) 254:113888. doi: 10.1016/j.physbeh.2022.113888, PMID: 35728627

[63] Speed CA Campbell R. Mechanisms of strength gain in a handgrip exercise programme in rheumatoid arthritis. Rheumatol Int. (2012) 32:159–63. doi: 10.1007/s00296-010-1596-x, PMID: 20697896

[64] Stojanović MDM Mikić MJ Milošević Z Vuković J Jezdimirović T Vučetić V. Effects of chair-based, low-load elastic band resistance training on functional fitness and metabolic biomarkers in older women. J Sports Sci Med. (2021) 20:133–41. doi: 10.52082/jssm.2021.133, PMID: 33707996 PMC7919366

[65] Marotta N Demeco A Indino A De Scorpio G Moggio L Ammendolia A. Nintendo Wiitm versus Xbox Kinecttm for functional locomotion in people with Parkinson's disease: a systematic review and network meta-analysis. Disabil Rehabil. (2022) 44:331–6. doi: 10.1080/09638288.2020.1768301, PMID: 32478581

[66] Benton MJ Spicher JM Silva-Smith AL. Validity and reliability of handgrip dynamometry in older adults: a comparison of two widely used dynamometers. PLoS One. (2022) 17:e0270132. doi: 10.1371/journal.pone.0270132, PMID: 35727792 PMC9212147

[67] Wu Y Wang W Liu T Zhang D. Association of grip strength with risk of all-cause mortality, cardiovascular diseases, and cancer in community-dwelling populations: a meta-analysis of prospective cohort studies. J Am Med Dir Assoc. (2017) 18:551.e17–35. doi: 10.1016/j.jamda.2017.03.011, PMID: 28549705

[68] Van Driessche S Van Roie E Vanwanseele B Delecluse C. Test-retest reliability of knee extensor rate of velocity and power development in older adults using the isotonic mode on a Biodex system 3 dynamometer. PLoS One. (2018) 13:e0196838. doi: 10.1371/journal.pone.0196838, PMID: 29723252 PMC5933798

[69] De Oliveira SN Leonel L Sudatti Delevatti R Heberle I Moro ARP. Effect of elastic resistance training on functional capacity in older adults: a systematic review with meta-analysis. Physiother Theory Pract. (2022) 39:2553–68. doi: 10.1080/09593985.2022.2085219 35652939

[70] Sato K Kuroki K Saiki S Nagatomi R. Improving walking, muscle strength, and balance in the elderly with an Exergame using Kinect: a randomized controlled trial. Games Health J. (2015) 4:161–7. doi: 10.1089/g4h.2014.0057, PMID: 26182059

[71] Rikli RE Jones CJ. Development and validation of criterion-referenced clinically relevant fitness standards for maintaining physical independence in later years. Gerontologist. (2013) 53:255–67. doi: 10.1093/geront/gns071, PMID: 22613940

[72] Bohannon RW. Daily sit-to-stands performed by adults: a systematic review. J Phys Ther Sci. (2015) 27:939–42. doi: 10.1589/jpts.27.939 25931764 PMC4395748

[73] Özkeskin M Özden F Ar E Yüceyar N. The reliability and validity of the 30-second chair stand test and modified four square step test in persons with multiple sclerosis. Physiother Theory Pract. (2022) 39:2189–95. doi: 10.1080/09593985.2022.2070811 35471847

[74] Martis R Crowther CA Shepherd E Alsweiler J Downie MR Brown J. Treatments for women with gestational diabetes mellitus: an overview of Cochrane systematic reviews. Cochrane Database Syst Rev. (2018) 8:Cd012327. doi: 10.1002/14651858.CD012327.pub2 30103263 PMC6513179

[75] El-Kotob R Ponzano M Chaput JP Janssen I Kho ME Poitras VJ . Resistance training and health in adults: an overview of systematic reviews. Appl Physiol Nutr Metab. (2020) 45:S165–s179. doi: 10.1139/apnm-2020-0245, PMID: 33054335

[76] Leung LYL Tam HL Ho JKM. Effectiveness of tai chi on older adults: A systematic review of systematic reviews with re-meta-analysis. Arch Gerontol Geriatr. (2022) 103:104796. doi: 10.1016/j.archger.2022.104796, PMID: 36058045

[77] Shen Y Shi Q Nong K Li S Yue J Huang J . Exercise for sarcopenia in older people: a systematic review and network meta‐analysis. J Cachexia Sarcopenia Muscle. (2023) 14:1199–211. doi: 10.1002/jcsm.13225 37057640 PMC10235889

[78] Borg GA. Psychophysical bases of perceived exertion. Med Sci Sports Exerc. (1982) 14:377–81. PMID: 7154893

[79] Santos A Lonsdale C Lubans D Vasconcellos D Kapsal N Vis-Dunbar M . Rates of compliance and adherence to high-intensity interval training in insufficiently active adults: a systematic review and meta-analysis protocol. Syst Rev. (2020) 9:56. doi: 10.1186/s13643-020-01301-0, PMID: 32183892 PMC7077158

[80] Freed SA Sprague BN Stephan AT Doyle CE Tian J Phillips CB . Feasibility and enjoyment of exercise video games in older adults. Front Public Health. (2021) 9:751289. doi: 10.3389/fpubh.2021.751289, PMID: 34805074 PMC8602072

[81] Afonso J Ramirez-Campillo R Clemente FM Büttner FC Andrade R. The perils of misinterpreting and misusing “publication Bias” in meta-analyses: an education review on funnel plot-based methods. Sports Med. (2023). doi: 10.1007/s40279-023-01927-9 PMC10933152 37684502

[82] Garcia-Alamino JM Bankhead C Heneghan C Pidduck N Perera R. Impact of heterogeneity and effect size on the estimation of the optimal information size: analysis of recently published meta-analyses. BMJ Open. (2017) 7:e015888. doi: 10.1136/bmjopen-2017-015888 PMC5695413 29122784

[83] Higgins JPT Li T. Exploring heterogeneity. System Rev Health Res. (2022). doi: 10.1002/9781119099369.ch10

